# The effect of fruit smoothie supplementation on psychological distress and biomarkers among people with opioid dependence receiving opioid agonist therapy: a randomized controlled trial

**DOI:** 10.1186/s12916-025-04347-w

**Published:** 2025-08-29

**Authors:** Elaheh Javadi Arjmand, Lise Margrete Thomassen, Karl Trygve Druckrey-Fiskaaen, Einar Furulund, Jørn Henrik Vold, Tesfaye Madebo, Rune Blomhoff, Jan Tore Daltveit, Hege Berg Henriksen, Fatemeh Chalabianloo, Kjell Arne Johansen, Torgeir Gilje Lid, Lars Thore Fadnes, the ATLAS4LAR study group, Vibeke Bråthen Buljovcic, Siv-Elin Leirvåg Carlsen, Jan Tore Daltveit, Tine Berger Edvardsdal, Karl Trygve Druckrey-Fiskaaen, Lars T. Fadnes, Trude Fondenes, Per Gundersen, Anne Eriksen Hammer, Else-Marie Løberg, Mette Hegland Nordbotn, Maria Olsvold, Marianne Cook Pierron, Kristin Sannerud, Christine Sundal, Beate Haga Trettenes, Jørn-Henrik Vold, Maren Borsheim Bergsaker, Tine Selmer Cruickshank, Eivin Dahl, Tone Lise Eielsen, Torhild Fiskå, Einar Furulund, Eirik Holder, Torgeir Gilje Lid, Tesfaye Madebo, Mari Soot, Rune Blomhoff, Hege Berg Henriksen

**Affiliations:** 1 Bergen Addiction Research and the Norwegian Research Center for Agonist Treatment of Substance Use Disorders, Department of Addiction Medicine, Haukeland University Hospital, Bergen, Norway; 2 Department of Global Public Health and Primary Care, University of Bergen, Bergen, Norway; 3 Centre for Alcohol and Drug Research, Stavanger University Hospital, Stavanger, Norway; 4 Oral Health Centre of Expertise Rogaland, Stavanger, Norway; 5 Division of Psychiatry, Haukeland University Hospital, Bergen, Norway; 6 Department of Respiratory Medicine, Stavanger University Hospital, Stavanger, Norway; 7 Department of Clinical Science, University of Bergen, Bergen, Norway; 8 Department of Nutrition, Institute of Basic Medical Sciences, University of Oslo, Oslo, Norway; 9 Department of Clinical Service, Division of Cancer Medicine, Oslo University Hospital, Oslo, Norway; 10 Department of Health Services, Norwegian Institute of Public Health, Oslo, Norway; 11 Faculty for Health Sciences, University of Stavanger, Stavanger, Norway

**Keywords:** Randomized controlled trial, Fruit, Food supplementations, Opioid agonist therapy, Substance use disorder, Psychological distress

## Abstract

**Background:**

Unhealthy diets are common among individuals with opioid dependence. While fruit- and vegetable-rich diets have shown mental health benefits, evidence is limited for those receiving opioid agonist therapy (OAT). This trial evaluated the effectiveness of fruit smoothie supplementation for people receiving OAT compared to standard treatment without fruit smoothie supplementation.

**Methods:**

In this multicenter randomized controlled trial (FruktBAR), 311 participants receiving OAT were randomized (5:3 intervention:control) to receive either a daily 250 ml fruit smoothie for 16 weeks in addition to standard OAT or standard OAT alone. The primary outcome was the difference between the arms in changes in psychological distress, measured by the ten-item Hopkins Symptom Checklist (SCL-10%) from baseline to the end of the intervention. The secondary outcomes included changes in fatigue symptoms, measured using the three-item Fatigue Severity Scale, physical fitness, measured by a 4-min step test, carotenoid and folate biomarkers.

**Results:**

At baseline, 131 participants (70%) in the intervention arm and 91 (73%) in the control arm had a low intake of fruits and vegetables. In the intervention arm, the mean SCL-10% score at baseline was 43.9% (95% confidence interval (CI): 40.4, 47.4), which was reduced to 41.6% (CI: 38.0, 5.1) at the end of the trial. In the control arm, the mean SCL-10% score was 43.6% at baseline (CI: 39.3, 48.0) and decreased to 41.5% (CI: 37.1, 45.8) at the end of the trial period. No significant difference in the change of psychological distress between the intervention and control arms was found (− 0.14%; CI: − 4.49, 2.22). Additionally, no changes were found between the intervention and control arms regarding fatigue symptoms, physical fitness, carotenoid, or folate biomarkers. The mean consumed fruit smoothies reported in the intervention arm was 3.9 bottles per week (SD 1.5).

**Conclusions:**

Fruit smoothie supplementation over a 16-week period did not impact psychological distress, fatigue, physical fitness, carotenoids, or folate biomarkers among people receiving OAT. Although the smoothies were successfully delivered to the participants, our data indicates suboptimal adherence to the intervention rather than the lack of efficacy.

**Trial registration:**

ClinicalTrials.gov NCT05229770. Registered on 08 February 2022.

**Supplementary Information:**

The online version contains supplementary material available at 10.1186/s12916-025-04347-w.

## Background

Substance use disorder (SUD) is linked to various adverse effects on psychological, social, and physical well-being [1, 2]. Compared to the general population, people who use illicit drugs have a higher risk of non-communicable diseases such as pulmonary diseases, cardiovascular diseases, psychological disorders, and premature death [2–4]. 

Psychiatric disorders and SUD are highly associated disorders [5, 6], which may contribute to complex medical and psychosocial challenges [7]. This co-morbidity is linked to lower quality of life and worse psychosocial and physical health, which highlights the need for addressing mental health in SUDs [8, 9]. A prospective cohort study that investigated the prevalence and changes in mental health symptoms among the SUD population found a considerable burden of mental health symptoms in these patients [10].

Psychological distress is a general term that covers a variety of common symptoms of psychiatric disorders [11]. Various studies have shown an association between psychological distress and eating behaviors, where feeling stressed triggers individuals towards eating more or eating high-sugar food items [12, 13]. Considering the high prevalence of psychological distress among people with SUD, such unhealthy eating habits are common, both during active drug use and under treatment [14, 15]. During recovery, there are several obstacles to maintaining a healthy diet, including food insecurity and lack of access to healthy food choices, poor oral health, and impaired saliva secretion, which makes it painful to chew [16–18]. Moreover, chronic substance use can change the regular appetite rhythm and prioritize the substance, gradually becoming the primary neurochemical reward superseding food and drinks [19]. This can lead to impulsive eating behaviors, seeking easy-to-digest food choices such as high-sugar foods [19]. Other examples of unhealthy dietary habits among people with SUDs are skipping meals, fasting to prolong the effect of the substance, and low intake of fruits and vegetables [16, 20, 21].

Previous studies, including two trials among people with depression in other contexts, have reported encouraging results on the mental health benefits, e.g., reduction in depression symptoms, from diets rich in fruit and vegetables [22–24]. One of the suggested pathways of how the intake of fruits and vegetables can affect mental health is related to the antioxidant activity of carotenoids [25, 26]. Carotenoids are found in high concentrations in fruits and vegetables. The most abundant carotenoids found in human plasma are β-carotene, α-carotene, β-cryptoxanthin, lycopene, lutein, and zeaxanthin [27]. A trial among male tobacco smokers reported altered gene expressions associated with cellular protective mechanisms following an eight-week intervention with kiwi or antioxidant-rich meals [28]. In addition to their biological actions in the human body, carotenoids, together with folate, are known biomarkers of fruit and vegetable intake [25, 29]. Folate is an essential micronutrient and an important co-factor in one-carbon metabolism—regulating cellular functions, including DNA synthesis [30]. Adequate folate status has been found to be important in maintaining good mental health, and a deficiency in plasma folate is known to impair cognitive functions [30, 31]. In a cohort study investigating folate status among people with SUD receiving opioid agonist treatment (OAT), low folate status was identified in 48% of the study population [32]. The researchers reported that the severity of substance use, injection of substances, and OAT medication dosage were associated with a reduction in serum folate over time [32]. To our knowledge, no previous study has investigated blood carotenoid levels in people with SUDs.

Although diets rich in fruits and vegetables have been associated with improved mental health in the general population and among people with depression, no large-scale trials have, to our knowledge, investigated such interventions among individuals with SUD, particularly those with opioid dependence [15]. The aim of this trial was to investigate the effect of an integrated fruit smoothie supplementation on the levels of psychological distress, as well as fatigue severity, physical fitness, folate, and carotenoid biomarkers among patients receiving OAT.

## Methods

### Study design and setting

This study was a multicenter individually randomized controlled trial of a dietary intervention for patients with opioid dependence receiving OAT. A protocol paper and extended analysis plan have been published [33, 34]. The target population included people diagnosed with opioid dependence according to the ICD-10 [35]. Participants were recruited from seven outpatient OAT clinics in Bergen and Stavanger in the period from April 2022 to November 2023. A pilot study by our team involving a similar intervention has demonstrated favorable experiences [36]. Approval for the study was received from the regional ethical committee (no. 155386/REK Sør-Øst -B, dated 23.09.2020/03.12.2021/05.04.2022). Written informed consent was obtained from all participants prior to enrolment. Results are reported in compliance with the Consolidated Standards of Reporting Trials (CONSORT) guidelines, which were also extended to non-pharmacologic treatments [37].

### Participants

This study is part of the ATLAS4LAR project, which aimed to improve well-being among patients receiving OAT through the integration of additional interventions [38]. Patients who completed the annual health assessment were considered eligible and were subsequently screened according to the study’s inclusion and exclusion criteria.

#### Inclusion criteria

Eligibility criteria were patients receiving at least weekly OAT medication at the OAT outpatient clinic, ≥ 18 years or older, providing informed consent, and consuming < 5 servings per day of fruits and vegetables.

#### Exclusion criteria

Participants were deemed ineligible if they had allergies or a history of anaphylactic reactions to any of the fruits and vegetables used in the intervention, or if they had a history of poorly regulated diabetes type 1 or 2, as defined by national guidelines (indicated by glycated hemoglobin levels ≥ 54 mmol/mol) [39].

**Fig. 1:**
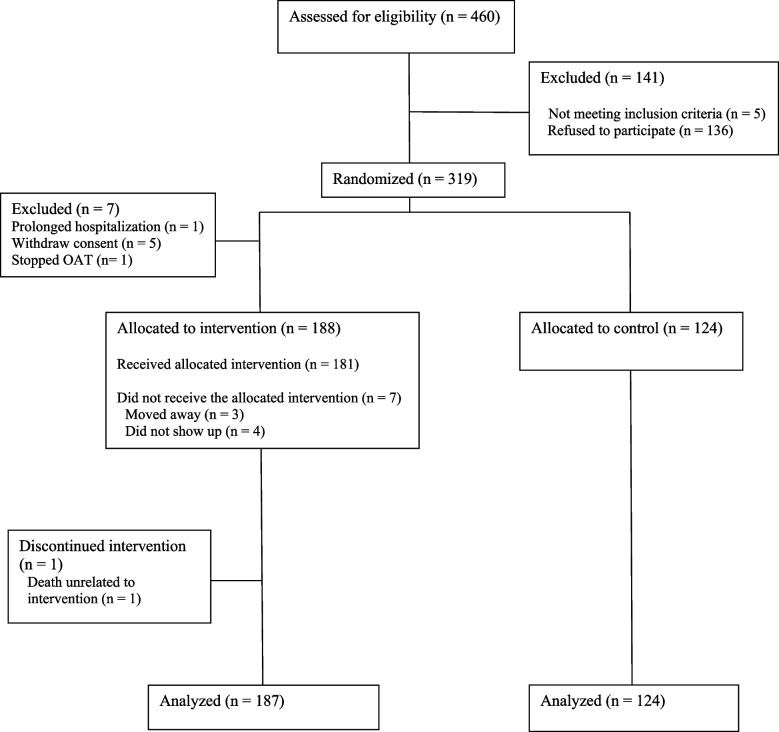
CONSORT flow diagram for the trial participants

We assessed 460 people for eligibility; among them, 141 were excluded. Consequently, 319 participants were randomized into either the intervention arm (195) or the control arm (124) (Fig. 1). Data from 187 participants in the intervention group and 124 participants in the control group were included in the analysis.

### Randomization and masking

Randomization codes with block sizes of eight were used in a 5:3 ratio between intervention and control arms (changed from 1:1 in the initial protocol to 5:3 in the revised protocol) [34]. We used the data entry program CheckWare (CheckWare, Trondheim, Norway) to assign an automatic, hidden number to each participant. Only one of the researchers (LTF), not involved in data collection, had access to this list, in addition to a randomization assistant who managed the assignment. This arrangement enabled the concealment of the randomization algorithm.

### Blinding

Although full blinding of the participants was not feasible, several strategies were used to reduce the risk of bias. The analyst (JHV) was blinded to group allocation until just before submission of the manuscript, and the study nurses who conducted measurements were instructed not to record the randomization status of the participants (although they had access to this information). Furthermore, participants in the intervention arm were not informed of the details of follow-up in the control arm and vice versa.

### Intervention

#### Intervention arm

The integrated fruit smoothie intervention consisted of a 250-ml fruit smoothie as a daily supplement for 16 weeks. The fruit smoothies were marketed products with a combination of fruits, including apples, pineapples, mangos, bananas, oranges, blueberries, passion fruits, coconut, lime, and blackcurrant. The nutrient content of the fruit smoothies is presented in Additional file 1: Table S1. The participants received a mixture of different smoothies, and they could choose to change the mixture based on their preferences. During weekly intervention sessions, the staff at OAT clinics provided the participants in the intervention arm with smoothie bottles on top of their standard OAT medication for the upcoming week with an oral agreement to consume one bottle per day. They were given the option to either come to the clinic to pick up the smoothies or have them delivered along with their opioid agonist therapy medication. We measured the adherence to the study protocol by the number of fulfilled study visits; in other words, the number of delivered smoothie bottles to the participants.

#### Standard arm

The control arm received their standard OAT treatment, including psychosocial support and opioid agonists such as buprenorphine or methadone, similar to the intervention group, without any dietary interventions and did not receive fruit smoothie supplementation (Additional file 1: Text S2).

### Outcomes and assessments

Study nurses assessed participants in both arms for the primary and secondary outcomes at the start of the intervention (baseline) and program completion (12–16 weeks) (Additional Text 1). The primary outcome was changes in psychological distress. Psychological distress was assessed using the validated Norwegian translation of the ten-item version of the Hopkins Symptom Checklist with percentage scaling (SCL-10%) at baseline and the end of intervention (EOI) (weeks 12–16) [40]. The SCL-10% includes 10 items (Additional file 1: Text S1), each evaluated on a four-point scale ranging from not bothered at all (item score = 1) to extremely bothered (item score = 4). The mean item score was calculated by taking the sum of the scores and dividing it by the number of items collected. The cut-off for high psychological distress was the mean SCL-10 score equal to or more than 1.85 [40]. The mean SCL-10 was re-scaled to a percentage scale (0 indicating no psychological distress and 100 indicating maximum psychological distress) with the cut-off at 28% for substantial psychological distress, indicating likely mental health disorders.

#### Secondary outcomes

Changes in fatigue symptoms were assessed using the 3-item Fatigue Severity Scale (FSS-3) [41]. Each item was rated on a Likert scale ranging from 1 (no fatigue) to 7 (worst fatigue), and the scores were summed to represent the overall fatigue level. Physical fitness was assessed using the 4-min step test, with changes in the number of repetition cycles serving as the outcome measure (Additional file 1: Text S1) [42]. Folate and carotenoids were reported as indicators of fruit smoothie intake and potential health effects and were compared between the arms. Folate levels were analyzed for all participants, while carotenoid levels were assessed in a random subsample comprising approximately one-quarter of the participants (control: *n* = 27, intervention: *n* = 49). Both folate and carotenoid concentrations were reported in nanomoles per liter (nmol/L). According to the World Health Organization, the cut-off level for clinical low serum folate is 13.5 nmol/L, which also serves as the threshold for recommending dietary advice or supplementation [43].

#### Blood sample collection

Non-fasting venous blood samples were collected from all study participants at baseline and the end of the intervention. The samples were transferred to the Department of Medical Biochemistry and Pharmacology at Haukeland University Hospital, where they were analyzed for folate in serum using electrochemiluminescence immunoassay. In addition, non-fasting dried blood spots were collected from a random subgroup (*n* = 76) of a quarter of the study participants at baseline and the end of the intervention (Additional file 1: Text S1).

### Analyses

#### Sample size

The sample size was calculated by the following assumptions: an allocation ratio of 5:3 (intervention:control), alpha of 0.05, two-sided test, 90% power, mean SCL-10 score in the control arm of 2.2 (SD: 0.8), and mean SCL-10 item score in the intervention arm of 1.9 (SD: 0.8). This would require a total of 323 individuals, 202 in the intervention and 121 in the control arms.

#### Statistical analyses

The intention-to-treat analysis is presented, where all participants are included based on randomization. Per-protocol analysis is also presented, where the participants receiving less than 75% of the fruit smoothie supplementation were excluded from the analyses. The statistical significance threshold was set to *p* < 0.05. We performed a linear mixed model to evaluate the change in SCL-10 scores following the intervention, considering a random intercept fixed slope model. The estimator was set to restricted maximum likelihood. Subgroup analysis was performed for the following factors: age, sex, level of intake of fruits and vegetables, living alone, and overall substance use score, as well as for individual substances (including opioids, alcohol, stimulants, benzodiazepines, and cannabis) (Additional file 1: Text S1). The same model was performed for subgroup analyses and biomarkers, stratified for each group. Continuous variables with a normal distribution are presented as mean (95% confidence intervals), while continuous variables not following the normal distribution are presented with median and interquartile range (IQR). Categorical values were presented with percentages. When the outcome value was missing at EOI, the value was set equal to the baseline. Further, sensitivity analysis was done for intention-to-treat and per-protocol analyses by removing the participants with missing EOI outcomes and repeating the analysis (Additional file 1: Text S2). We used Stata/SE17 (StataCorp, TX, USA) for the statistical analysis and R Studio (version 2024.04.02 + 764) to create and visualize the results for the biomarkers and subgroup analysis.

## Results

### Baseline demographics

There were no significant differences in baseline characteristics between participants in the intervention and control groups (Table 1). Most of the participants were males (60%), with a mean age of 47 years. Regarding OAT, 46% of the participants were receiving buprenorphine, and they frequently were using illicit substances like cannabis (42%), alcohol (50%), benzodiazepines (63%), and amphetamine or cocaine (47%). The intake of fruits and vegetables was low in both groups at baseline (73% in the control and 70% in the intervention group). A total of 69% of the participants in the control group and 70% in the intervention group had a high level of psychological distress scores (≥ 1.85) at baseline.

**Table 1:** Baseline characteristics and summary statistics (*n* (%))

	Control	Intervention
	(*N* = 124)	(*N* = 187)
Sex, *n* (%)		
Males	87 (70%)	135 (71%)
Age, mean (SD)	48 (11)	47 (12)
Body mass index mean (SD)	27 (6)	26 (6)
Education level		
< 10 years	11 (38%)	29 (56%)
≥ 10 years	18 (62%)	23 (44%)
Living alone, *n* (%)	80 (66%)	133 (71%)
Income, *n* (%)		
Paid work	5 (4%)	2 (1%)
Other income	117 (96%)	185 (99%)
OAT treatment, *n* (%)		
Methadone or others	57 (47%)	85 (46%)
Buprenorphine	65 (53%)	101 (54%)
Any substance use last 30 days, *n* (%) *		
Opioids	28 (23%)	46 (25%)
Alcohol	78 (63%)	92 (49%)
Amphetamines or cocaine	43 (35%)	62 (33%)
Benzodiazepines	64 (52%)	90 (48%)
Cannabis	92 (75%)	113 (60%)
Any injection of tablets/mixtures, *n* (%) *	9 (9%)	20 (13%)
Intake of fruits and vegetables, *n* (%)		
Low (< 2 servings per day)	93 (76%)	128 (67%)
Moderate (≥ 2 and < 4 servings per day)	22 (19%)	52 (29%)
High (≥ 4 servings per day)	8 (5%)	10 (4%)
High psychological distress, *n* (%) **	86 (69%)	133 (70%)
Fatigue severity score, mean (SD)	4.9 (2.1)	5.0 (2.1)
4-min step-test repetitions, mean (SD)	97.4 (34.1)	102.6 (43.1)

*Number of participants reporting substance use at least once per week for each of the substances. **Participants who had a mean SCL-10 item score of > 1.85

### The effect of fruit smoothie supplementation on psychological distress

The median of delivered smoothies was 88%, and 127 of the participants in the intervention arm received more than 75% of the smoothies (Additional file 1: Fig. S1). Among these, the mean self-reported consumption of smoothies per week was 3.9 bottles per week (SD 1.49) (Additional file 1: Fig. S2). The level of fruit and vegetable intake at the end of the intervention did not change for the control arm, while about two-fifths of the intervention arm moved from low to moderate intake category (Additional file 1: Fig. S3).

**Fig. 2:**
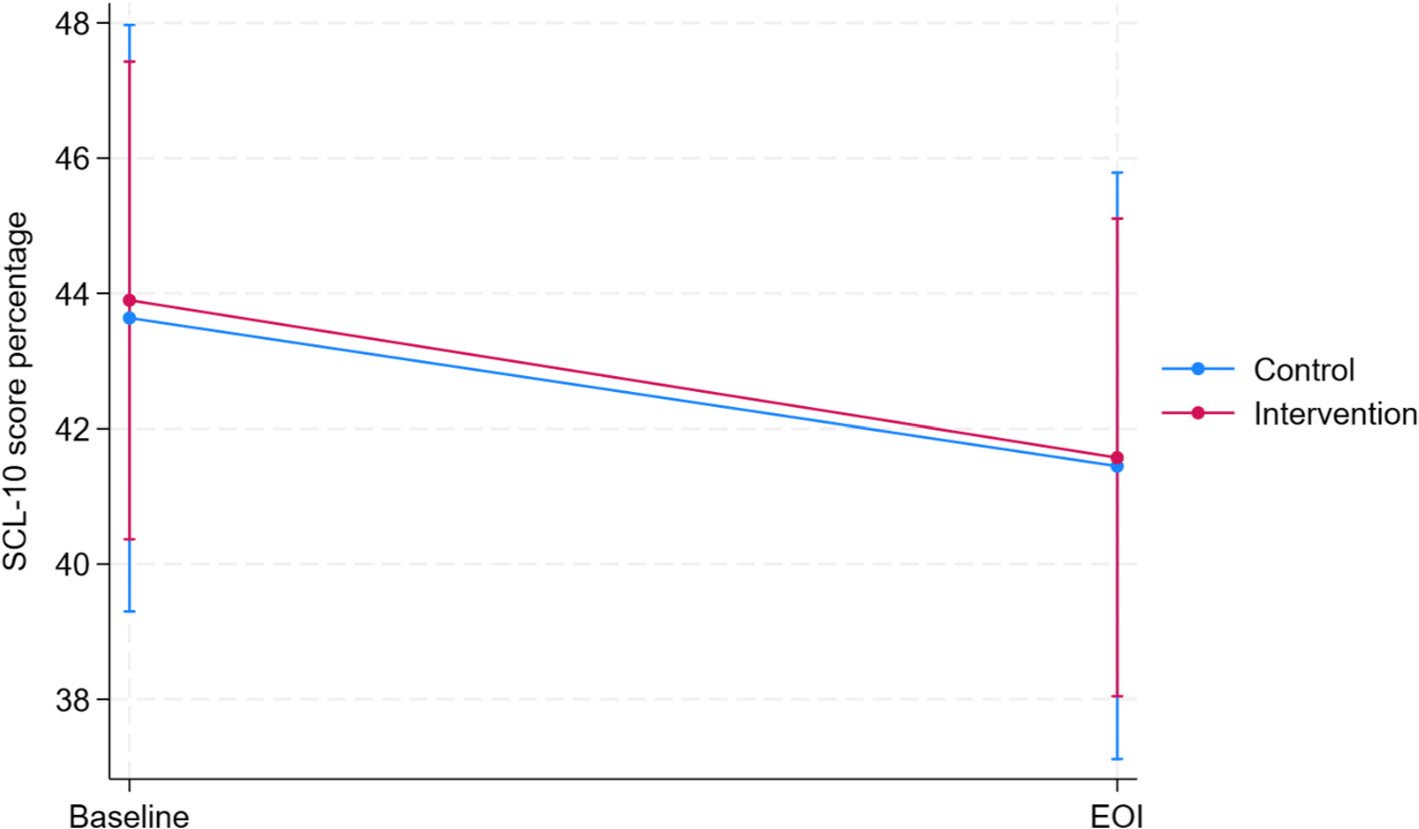
Changes in the mean SCL-10% scores from baseline to the end of intervention in intervention and control groups (based on intention-to-treat analysis). EOI, end of intervention

**Fig. 3:**
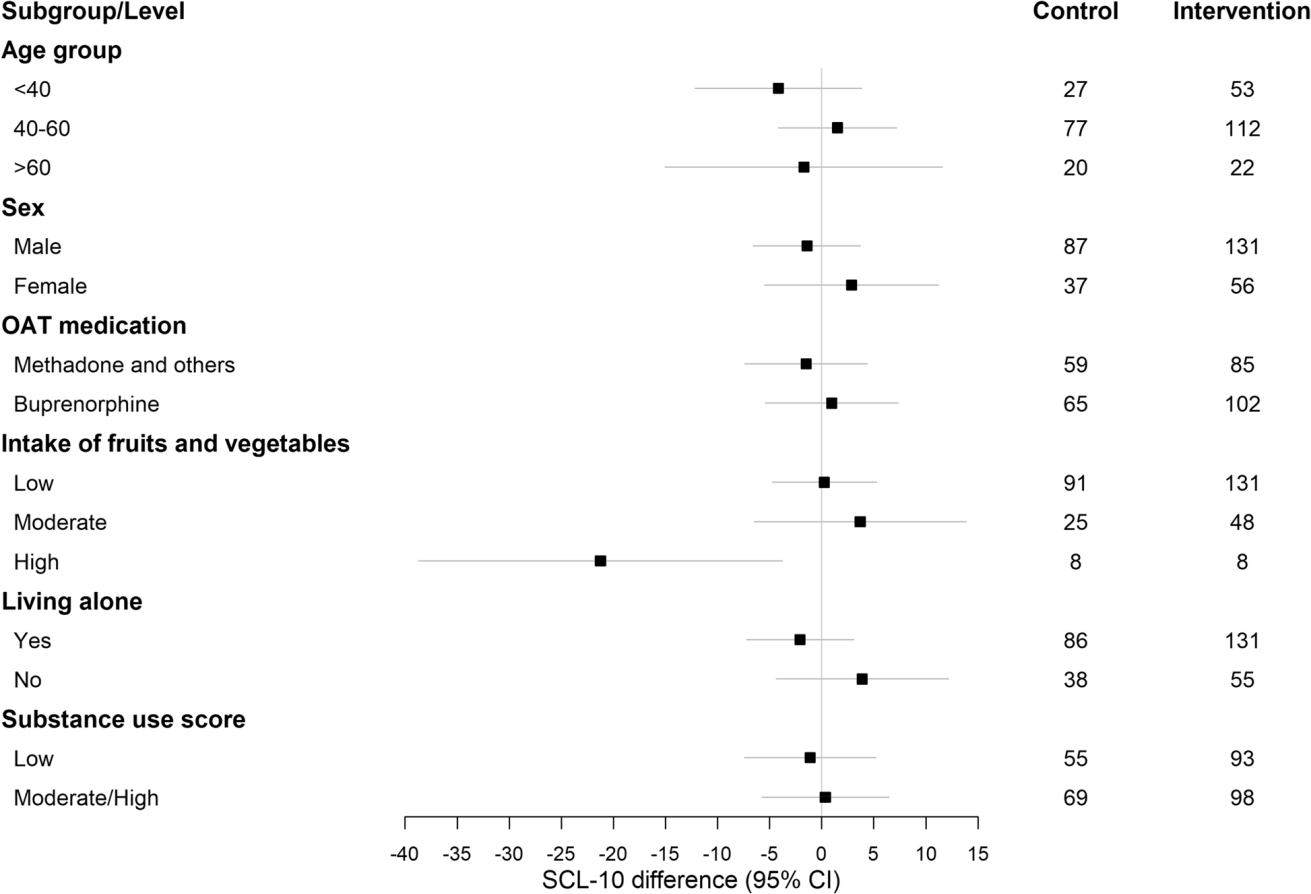
Forest plot of subgroup analysis. The difference between arms in changes in the percentage of mean SCL-10, stratified for age, sex, OAT medication, intake of fruits and vegetables, living alone, and substance use score. The changes in SCL-10% are estimated using linear mixed models

The mean SCL-10% score at baseline was 43.9% (95% CI: 40.4, 47.4) in the intervention arm and 43.6% (95% CI: 39.3, 48.0) in the control arm (Fig. 2, Table 2). At the EOI, the mean SCL-10% score was reduced to 41.6 (95% CI: 38.0, 45.1) in the intervention arm and 41.5 (95% CI: 37.1,45.8) in the control arm. Most of the patients in both arms were above the threshold for high levels of psychological distress at the end of the intervention period, while around one-fifth moved from the high to normal category (Additional file 1: Fig. S4). No difference in the change of SCL-10% between the arms was found over the intervention period (− 0.14%, 95% CI: − 4.49, 4.22). The per-protocol analysis showed similar results (Additional file 1: Fig. S5). Sensitivity analysis, where the participants with missing outcome values were removed, was in line with the main analysis (Additional file 1: Fig. S6 and S7). The subgroup analysis showed no significant differences in the outcome among different age groups, sex, type of OAT medication, living alone or not, substance use score, and use of different substances, including opioids, alcohol, stimulants, benzodiazepines, and cannabis (Fig. 3, Additional file 1: Fig. S8). In a small subgroup of those who had high intake of fruits and vegetables at baseline (*n* = 8 for the control group and *n* = 8 for the intervention group), there was a difference in changes of SCL-10% between the arms. However, this should be interpreted with caution and could be due to random variation.

**Table 2:** The psychological distress scores, carotenoids, and folate levels at the end of the intervention and the absolute difference between arms

Outcome*	Mean (95%CI) or median (IQR)		Change difference across arms (slope)** (with 95%CI)
	Intervention	Control	
Mean SCL-10% score (ITT)	41.6 (38.0, 45.1)	41.5 (37.1, 45.8)	− 0.1 (− 4.5, 4.2)
Mean SCL-10% score (PP)	40.2 (36.0, 44.5)	41.5 (37.1, 45.8)	0.6 (− 4.2, 5.5)
Sum FF3-S score (ITT)	4.8 (4.5, 5.1)	4.8 (4.5, 5.1)	− 0.1 (− 0.6, 0.4)
Sum FF3-S score (PP)	4.7 (4.3, 5.1)	4.8 (4.5, 5.1)	− 0.2 (− 0.8, 0.3)
4-min step-test repetitions (ITT)	103.0 (97.7, 108.4)	101.9 (95.4, 108.4)	− 4.1 (− 10.9, 2.7)
4-min step-test repetitions (PP)	103.3 (96.6, 109.9)	101.9 (95.1, 108.7)	− 4.4 (− 12.3, 3.6)
Folate, nM (ITT)	14.6 (18.0)	13.4 (17.2)	0.6 (− 1.8, 3.0)
Folate, nM (PP)	14.0 (18.3)	13.4 (17.2)	0.2 (− 2.5, 2.9)
Total carotenoid, nM (ITT)	226 (230)	212 (191)	24 (− 44, 92)
Total carotenoid, nM (PP)	272 (291)	212 (191)	50 (− 29, 129)
α-carotene, nM (ITT)	12.6 (11.4)	12.0 (14.2)	2 (− 1, 6)
α-carotene, nM (PP)	11.6 (14.7)	12.0 (14.2)	4 (− 0, 7)
β-carotene, nM (ITT)	58.8 (86.5)	57.8 (76.9)	15 (− 7, 36)
β-carotene, nM (PP)	63.5 (92.8)	57.8 (76.9)	23 (0, 47)
Lutein, nM (ITT)	26.4 (36.1)	27.6 (24.2)	− 1 (− 12, 9)
Lutein, nM (PP)	27.8 (35.4)	27.6 (24.2)	0 (− 11, 11)
Zeaxanthin, nM (ITT)	7.4 (7.1)	7.8 (5.3)	0 (− 2, 2)
Zeaxanthin, nM (PP)	7.4 (7.6)	7.8 (5.3)	1 (− 2, 3)
β-cryptoxanthin, nM (ITT)	13.9 (22.0)	12.6 (21.0)	2 (− 3, 8)
β-cryptoxanthin, nM (PP)	13.7 (23.5)	12.6 (21.0)	5 (− 3, 12)
Lycopene, nM (ITT)	76.3 (79.5)	90.0 (70.0)	0 (− 32, 32)
Lycopene, nM (PP)	76.8 (96.9)	90.0 (70.0)	9 (− 28, 47)

*Measured at the end of the trial period (12–16 weeks range) ** The absolute difference between arms was calculated using the linear mixed model. *ITT* intention to treat, *PP* per protocol, *SCL-10* Hopkin’s checklist symptom for psychological distress, *FSS-3* 3-item fatigue severity scale

### The effect of fruit smoothie supplementation on fatigue, physical fitness, and biomarkers

At the EOI, the sum FSS-3 score was slightly reduced in both arms, with no significant differences between the arms in changes of the sum FSS-3 score (− 0.1, 95% CI: − 0.6; 0.4) (Table 2, Additional file 1: Fig. S9). The mean number of repetitions of steps in the 4-min step test was slightly increased for both arms at EOI, with no significant difference between the arms (− 4.1, 95% CI: − 10.9; 2.7) (Table 2, Additional file 1: Fig. S10). The per-protocol analysis showed similar results (Additional file 1: Figs. S11 and S12).

**Fig. 4:**
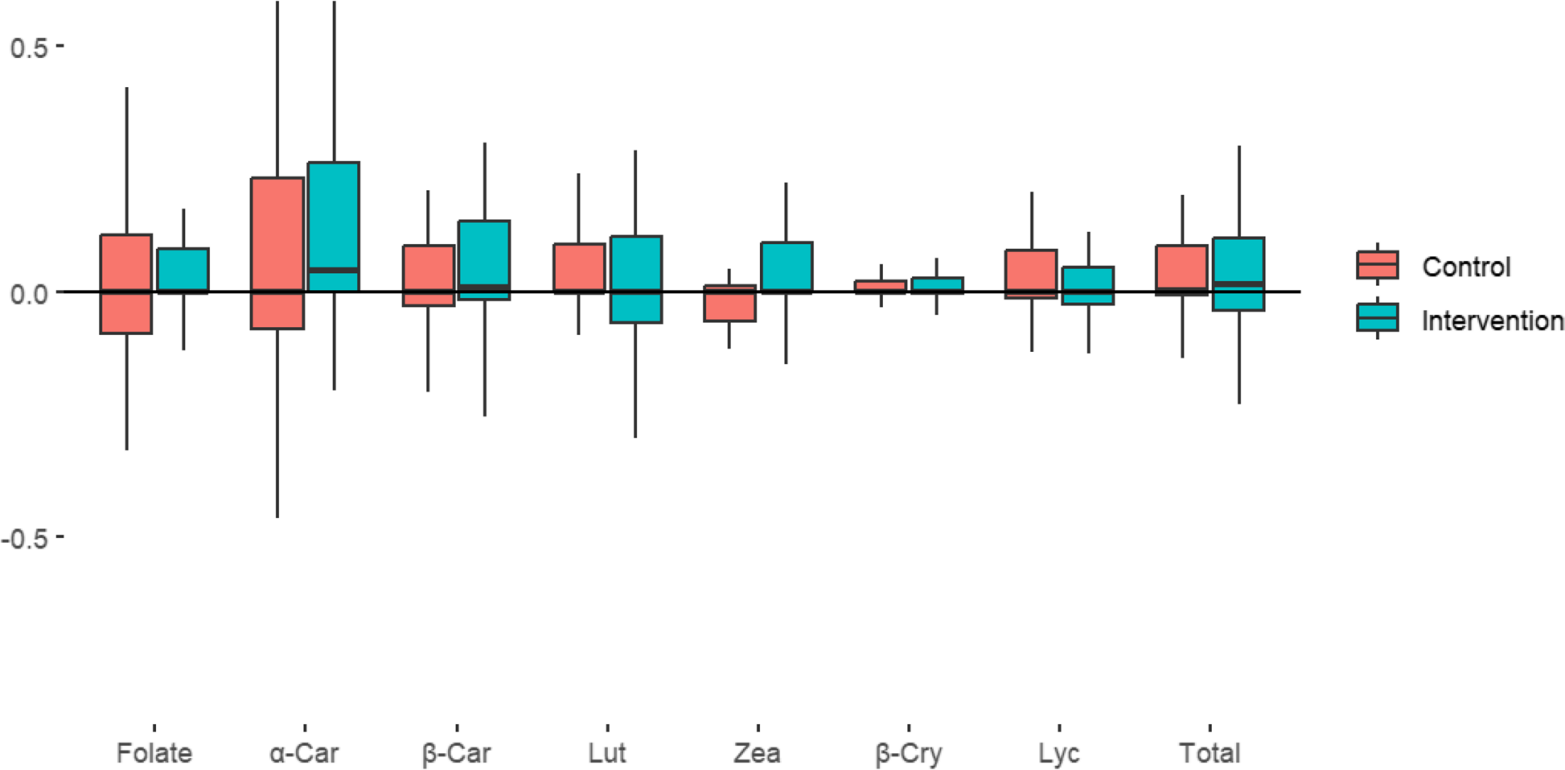
The changes in median levels of biomarkers of fruit smoothie intake after the intervention. The ranges have been standardized by dividing each value by the largest estimate within that measurement. Total refers to total carotenoids. Folate: *n* = 311; carotenoids: *n* = 76

At baseline, the median folate level was 12.0 (SD 14.0) for the intervention group and 11 (SD 15) for the control group (Table S2). At EOI, the median folate levels were increased to 14.6 (SD 18.0) in the intervention arm and 13.4 (SD 17.2) in the control arm (Table 2). No significant differences between the arms in changes in the mean folate level were found (0.6, 95% CI: − 1.8, 3.0) (Fig. 4). For a random subgroup comprising one quarter of the participants with available samples for carotenoids, the median total carotenoid levels at baseline were 223 (SD 205) in the intervention arm and 204 (SD 209) in the control arm (Table S2). This increased to 272 (SD 291) in the intervention arm and 212 (191) in the control arm at the EOI. There were no significant differences between arms in the changes of the total carotenoid levels (50, 95% CI: − 29, 129). Further, there were no significant differences between the arms in changes in levels of subgroups of carotenoids such as α-carotene, β-carotene, lutein, zeaxanthin, β-cryptoxanthin, and lycopene. The per-protocol analysis showed the same results.

## Discussion

This randomized controlled trial assessed the effect of fruit smoothie supplementation for a period of 16 weeks on psychological distress and biomarkers of fruit and vegetable intake among patients receiving OAT. Although the psychological distress was reduced slightly in the intervention period, there were no clear differences between the intervention and control arms. Similarly, the fatigue severity score and levels of biomarkers of intake of fruit and vegetables were slightly enhanced at the end of the intervention period; however, there were no significant differences between the arms. No substantial changes were seen in the physical function test after the intervention.

Most participants exhibited high levels of psychological distress at baseline, consistent with findings from similar studies in Norway, which reported elevated distress levels among this population compared to the general population [10, 40]. This distress could be attributed to a combination of factors, including a difficult economic status and unstable housing and social situations [35]. It is possible that nutritional interventions effective in the general population may not sufficiently address the substantial psychological distress levels and variability introduced by distortions in housing situations and disrupted social relations. Several social determinants of mental health, beyond the nutritional aspects, are unlikely to change substantially during the intervention. This may limit the potential effect of fruit smoothies and other health-related interventions, as these aspects are highly important for how mental health is experienced [44]. The same argument could stand for fatigue severity, as the reasons related to their fatigue could be related to sociodemographic factors such as debt difficulties, which the intervention is less likely to be effective [45]. On the other hand, for measures of physical fitness, there was a substantial proportion of missing measurements, partly due to some aversion to such testing among the participants and also to the need to conduct some of these interviews in settings where physical activity measurements were challenging.

The change in total carotenoid concentration in blood was the main outcome of the data analysis of biomarkers, and it was hypothesized that the fruit smoothie intervention would be reflected by an increase in total carotenoid in plasma among participants in the intervention arm compared to the control arm. The definition of a normal range for plasma levels of carotenoids varies between countries [46]. A study in Norway that assessed the plasma carotenoid levels divided by the intake of fruits reported mean total carotenoids were 1214 nmol/L in the group with a low intake (two portions per day) and 1561 nmol/L in the recommended intake (five portions per day) group [29]. The baseline levels in our sample were much lower (204 nmol/L for the control arm and 223 nmol/L for the intervention). Although no significant results were found for any of the biomarkers, we did see a non-significant increase in the concentration of total carotenoids in the intervention arm compared to the control arm. Most participants received more than 75% of the fruit smoothies, and among them, an increase in total carotenoids was 28% higher compared to the control arm. However, we would expect the increase to be higher if they were consumed as recommended. Our self-reported data on adherence indicates that about half of the smoothies in the intervention were consumed. This implies that the smoothies were not consumed daily. Thus, our data from both self-reported data and the biomarker data indicate that the level of adherence was suboptimal. We measured the adherence by the number of fruit smoothie bottles delivered to the participants on a weekly basis, but they might have been consumed in a single day or even shared with other people including persons in the control arm, as many of the participants in this trial were sharing housing. It is also possible that the duration was too short to provide sufficient effect, and potentially, a longer duration might have provided different results. Moreover, it is likely that this sample size was too small to have enough power to detect a true difference between the arms as only a subgroup of 76 participants was randomized to the carotenoid biomarker assessment. However, these biomarkers are not specific for fruit smoothies but give a general reflection of fruit and vegetable intake. A general concern for many nutritional trials is that it is not possible to blind the intervention, and participants in the control arm understand that they are not receiving the intervention believed to provide a health benefit. It is therefore possible that participants in the control arm were motivated to alter their eating behavior by including more fruits and vegetables in their diet.

In substance use treatment settings, nutritional implications are often neglected [19, 47]. Although this intervention showed limited impact on psychological distress, we argue that integrating more comprehensive and long-term nutritional strategies—alongside other lifestyle intervention—into standard OAT can be beneficial for these patients. Such integrated interventions align with harm reduction policies targeting SUD populations [48]. Our results are applicable to people with substance use disorders receiving OAT with or without well-controlled type 2 diabetes. Further interventions with a longer period and larger sample could shed light on the other possible benefits of nutritional supplementation among people with SUD.

### Limitations

First, the 16-week duration could have been insufficient to provide sufficient changes in the main outcomes, particularly in such a context where many experienced complex life struggles, including financial difficulties, stigma, social exclusion, being threatened, and many had health-related sequela from substance use. Second, the SCL-10 is not a diagnostic instrument for mental health disorders and cannot substitute for clinical interviews and more thorough psychiatric assessments in individuals with SUDs. Studies have shown that the SCL-10 is a better predictor for depression and anxiety compared to diagnostic tools, with approximately 50–60% of patients exhibiting symptoms of mental disorders qualifying for at least one or more mental disorders upon clinical assessment [49]. Third, a 1:1 algorithm was initially planned, but due to some misunderstanding in the coding of the randomization algorithm, we ended up with a 5:3 allocation ratio, which is described in a preprint statistical analysis plan [34]. The analytic power was minimally reduced, but the analytic power in our trial was still close to 90%. Fourth, many of the variables used in this study, such as substance use, injection of tablets and mixtures, intake of fruits and vegetables, and fatigue, are self-reported and thus are prone to recall bias. Fifth, many of the participants reported consumption of alcohol, which could limit the absorption of the nutrients available in fruit smoothies, specifically folate [50]. Moreover, since carotenoids are fat-soluble, the first step of their absorption is dissolution in lipids in the meal, and the absence of lipids in the gut can limit their absorption [51]. Furthermore, this study was conducted in a high-income setting where national health care covers most of the costs of the OAT and people with opioid dependence have access to social support; thus, the results cannot be generalized to low and middle-income settings. The psychosocial support, as part of the standard treatment, provided to both the intervention and control group could have contributed to changes in dietary patterns, which could have reduced potential differences between the arms. Lastly, and importantly, our data also indicates suboptimal adherence to the intervention. Thus, our trial shows a lack of effectiveness for providing fruit smoothies on mental health and physical fitness, but not necessarily a lack of efficacy of fruit smoothies in improving these health outcomes if the smoothies had been consumed as recommended.

## Conclusions

This randomized controlled trial found no effectiveness of integrating a fruit smoothie delivery intervention on psychological distress, fatigue, or physical fitness among people receiving OAT. Both self-reported consumption data and biomarkers indicated suboptimal adherence to the intervention. Therefore, the lack of observed improvements in mental health symptoms or physical performance is likely attributable to suboptimal adherence rather than a lack of efficacy from fruit smoothie supplementation. 

## Supplementary Information

Additional file 1: Text S1-S2, Figures S1-S12, Table S1-S2. Text S1- Questionnaire items. Table S1-Nutrient content in smoothie bottles. Text S2- Standard treatment and data handling. Figure S1- Delivery frequency. Figure S2 – Consumption pattern in intervention. Figure S3 – Changes in fruits and vegetables intake. Figure S4 – Changes in categories of psychological distress. Figure S5 – Per protocol analysis of psychological distress. Figure S6 – Sensitivity analysis of intention to treat. Figure S7—Sensitivity analysis of per protocol. Figure S8 – Forest plot of subgroup analysis. Figure S9 – Intention-to-treat analysis for fatigue score. Figure S10—Intention-to-treat analysis for physical fitness. Figure S11 – Per protocol analysis for fatigue score. Figure S12 – Per protocol analysis for physical fitness. Table S2 – Baseline values for secondary outcome

## Data Availability

The data supporting the findings of this study are not publicly accessible due to sensitivity concerns; however, anonymous data can be available from the corresponding author upon reasonable request. Data are in controlled-access data storage at Haukeland University Hospital.

## Abbreviations

SUD: Substance use disorder
OAT: Opioid agonist therapy
EOI: End of intervention
