# Supplementary material for: The effect of fruit smoothie supplementation on psychological distress and biomarkers among people with opioid dependence receiving opioid agonist therapy: a randomized controlled trial
Source: BMC Med. 2025 Aug 29;23:505. doi: 10.1186/s12916-025-04347-w (PMC12395640; doi:10.1186/s12916-025-04347-w)
Supplement: Supplementary file 1 — Additional file 1: Text S1-S2, Figures S1-S12, Table S1-S2. Text S1- Questionnaire items. Table S1-Nutrient content in smoothie bottles. Text S2- Standard treatment and data handling. Figure S1- Delivery frequency. Figure S2 – Consumption pattern in intervention. Figure S3 – Changes in fruits and vegetables intake. Figure S4 – Changes in categories of psychological distress. Figure S5 – Per protocol analysis of psychological distress. Figure S6 – Sensitivity analysis of intention to treat. Figure S7—Sensitivity analysis of per protocol. Figure S8 – Forest plot of subgroup analysis. Figure S9 – Intention-to-treat analysis for fatigue score. Figure S10—Intention-to-treat analysis for physical fitness. Figure S11 – Per protocol analysis for fatigue score. Figure S12 – Per protocol analysis for physical fitness. Table S2 – Baseline values for secondary outcome [file 12916_2025_4347_MOESM1_ESM.docx]

## Text S1:

## Assessment and outcomes, questionnaire items:

For sociodemographic factors, we extracted the data from the closest annual health assessment to the first study visit for each patient. Age was categorized into the following groups: <30 years, 30–39 years, 40–49 years, 50–59 years and ≥60 years. Income was categorized into paid work (full-time or part-time job) and other income (social benefits, daily allowance, sickness allowance or work clarification allowance, disability benefits, scholarship or grants, saved funds or inheritance, provided by others). OAT medication was categorized into methadone, buprenorphine, and other medications such as long-acting morphine.

Current substance use was assessed by asking the participants about the frequency of use of substances in the last 30 days. We reported any substance use or none.

Q: How often have you used the following substances in the past 30 days?

A: ‘Never’, ‘Less than 1 day/month’, ‘1-3 days/month’, ‘1-3 days/week’, ‘More than 3 days/week’, ‘More or less daily’

Q1: Alcohol

Q2: Cannabis (including marijuana, hash, cannabis oil)

Q3: Amphetamine (includes also methamphetamine, Phenmetrazine, khat, Betelnøtt, Ritalin, methylphenidate, Dexamin: speed)

Q4: Benzodiazepine (Sedative drugs and/or sleeping pills more than prescribed includig alprazolam (Xanor, Xanor dep.), diazepam (Stesolid, Valium, Vival, Apozepam), flunitrazepam (Rohypnol, Flunipam), clonazepam (Rivotril), lorazepam ( Ativan, Temesta), midazolam (Dormicum), nitrazepam (Apodorm, Mogadon), oxazepam (Sobril), temazepam, zolpidem (Stilnoct), zopiclone (Imovane, Zopiklone)).

Q5: Other opioids (for example: Over-the-counter pain relievers including Actiq, Aporex, buprenorphine, Codaxol, Cosylan, Dextropropoxyphene, Dolcontin, Durogesic, Ethylmorphine, fentanyl, Fortralin, hydrocodone, hydromorphone chloride, Kapanol, Ketalar, Ketamine, ketobemidone, Ketogan, Ketorax, codeine, methadone, morphine, morphine scopolamine, Nobligan, Norgesic, oxycodone, OxyContin, OxyNorm, Paralgin forte, Paralgin major, pentazocine, pethidine, Pinex forte, Suboxone, Subutex, Temgesic, tramadol, Tramgetic, opium)

Q6: Cocaine (including ‘crack’, ‘freebase’, ‘coca leaf’, ‘coca paste’)

The injection of substances was assessed by asking the participants how often they had injected crushed tablets or mixtures in the last 30 days. We reported categorizing this into any or none.

Q: Do you inject the following:

Crushed tablets

Liquid drugs as a mixture (Methadone, Vallergan, etc.) or rectal liquids (Stesolid etc.)

A: ‘Yes’, ‘No’

If Yes:

Q: How often do you inject?

A: ‘Less than 1 day/month’, ‘1-3 days/ month’, ‘1-3 days/week’, ‘More than 3 days/ week’, ‘Daily’

Intake of fruits and vegetables was categorized into low (less than two servings per day), moderate (two to four servings per day), and high (four and more servings per day).

Q: During the past month, how many portions of fruit and vegetables per day did you eat often?

A: 0-1-2-3-4-5-6-7-8-9-10 or more

Items focusing on psychological distress (Hopkins symptom checklist SCL-10):

Q: Have you experienced any of these problems last week (including today)?

A: ‘Not at all’, ‘A little’, ‘Quite a bit’ and ‘Extremely’

Q1: Suddenly scared for no reason

Q2: Feeling fearful

Q3: Faintness, dizziness, or weakness

Q4: Feeling tense or keyed up

Q5: Blaming yourself for things

Q6: Difficulty in falling asleep or staying asleep

Q7: Feeling of worthlessness

Q8: Feeling blue

Q9: Feeling hopeless about the future

Q10: Feeling everything is an effort

Items focusing on fatigue (three-item Fatigue Severity Scale FSS-3)

Q: In the last week, I have felt:

A: 1 ‘Strongly disagree’ 🡪 7 ‘Strongly agree’

I1: Fatigue causes frequent problems for me

I2: My fatigue prevents sustained physical functioning

I3: Fatigue interferes with carrying out certain duties and responsibilities

## 4-minute step-test

For evaluation of physical fitness, we used the 4-minute step test, where the participant stepped up and down in a 20 cm box for 4 minutes. The participants received the instructions as bellow:

“You will do a 4-minute step test, stepping on and off this 20 cm box as fast as you can. The goal is to complete as many steps as possible in 4 minutes. Make sure both feet are on top of the box before stepping down. You may slow down, stop, or sit if needed, but continue as soon as possible. I will notify you every minute.”

We used the number of steps in 4 minutes as the outcome (number of repetitions).

## Blood sample collection:

The non-fasting dried blood spots were collected by either capillary blood using a lancet to prick the finger or from a venous blood sample collected in an EDTA tube and transferred to the non-fasting dried blood spots paper using a DIFF-SAFE dispenser. Non-fasting dried blood spots papers were set to dry in a dark room for 2-4 hours before they were stored in the fridge at -20 degrees. All non-fasting dried blood spots papers were collectively shipped frozen to Vitas Analytical Services in Oslo, Norway, for analysis of carotenoids. Simultaneous quantification of carotenoids in non-fasting dried blood spots was performed using five 3.1mm punches eluted with water and extracted using an isopropanol-containing tool as an internal standard. The supernatant was injected into a high-performance liquid chromatography system coupled with a diode array detector and a fluorescence detector. Separation was performed on a reversed-phase C30 column.

**Table S1**: Nutrient content per 250 ml of the BAMA fruit smoothies.

|  | Mango and passion fruit | Pineapple and coconut | Blueberries and apple | Pineapple and mango |
| --- | --- | --- | --- | --- |
| Energy (kcal) | 125 | 160 | 133 | 118 |
| Fat (g) | 0.5 | 4.5 | 0.25 | 0.25 |
| Saturated fatty acids (g) | 0.25 | 4.3 | 0 | 0 |
| Carbohydrates (g) | 28 | 28 | 30 | 26 |
| Protein (g) | 1.25 | 1.5 | 1 | 1 |
| Vitamin A (µg-RE) | 22.5 | 2.5 | 5 | 17.5 |
| Vitamin E (mg-ATE) | 0.5 | 0.25 | 0.75 | 0.5 |
| Thiamine (mg) | 0.15 | 0.15 | 0.1 | 0.2 |
| Riboflavin (mg) | 0.3 | 0.13 | 0.3 | 0.15 |
| Niacin (mg) | 1.25 | 0.75 | 0.5 | 1 |
| Pyridoxine (mg) | 0.28 | 0.3 | 0.18 | 0.15 |
| Folate (µg) | 25 | 35 | 15 | 35 |
| Ascorbic Acid (mg) | 27.5 | 20 | 25 | 35 |

## Text S2

### Standard treatment

In Western Norway (The Department of Addiction Medicine at Haukeland University Hospital in Bergen and the Department of Substance Abuse and Addiction Treatment at Stavanger University Hospital in Stavanger), OAT is initially administered on an outpatient basis with supervised and observed intake of medications until an individualized dosage has been established. Then outpatient OAT clinics provide regular follow-ups by a multidisciplinary healthcare team consisting of physicians specializing in addiction medicine, nurses, psychologists, social workers, and peer counselors. This setting is a well-suited delivery platform to evaluate integrated interventions. Patients are administered methadone, buprenorphine, or morphine sulfate for opioid dependency as directly monitored therapy and as take-home medication. Patients are often offered between one and six take-home doses of the medication per week, based on factors such as concurrent drug usage, mental and physical co-morbidities, and attendance stability. During the COVID-19 pandemic medication was delivered at home by the OAT staff. In addition to OAT patients at the clinics, each has been assigned a therapist (usually a nurse or social worker) and is offered at least yearly health assessments by a physician and nurse. Persons in need of psychotherapy are referred to a psychologist at the OAT clinic. There is a close collaboration between the OAT-clinics and the patient's primary care physicians, municipal social workers, and municipal activities for persons with substance dependence.

### Data handling and sensitivity analysis

Baseline values were extracted from the health registry platform by matching the date of the start of the intervention with the date of the health assessment. For the end-of-intervention (EOI) values, the date for the last study visit was matched with the closest health assessment. When the outcome value was missing at EOI, the value was set equal to the baseline. Further, for intention-to-treat and per-protocol analyses, sensitivity analysis was done by removing the participants with missing EOI outcomes and repeating the analysis. For patients in the intervention group where the blood sample was collected >28 days after the end of the intervention, the EOT value was replaced with the baseline value (i.e. analyzed as no changes and interpreted conservatively).

For the 4-minute step test, the participants who took the test 2 months before or after the start of the intervention were removed from the analysis (n=56, 42 missed visits and 14 participants with pain or disability). If the data were missing at the EOI, it was replaced with baseline values (111 missing tests at EOI).

For the biomarkers, values under the lowest level of quantification (LLOQ) were replaced by the middle number between 0 and LLOQ. For the Carotenoid figure 3 in the article, relative values have been calculated. This was done by taking the absolute value and dividing by the highest number occurring for that variable.

Calculation of substance use score: Frequency of use of 5 substances were added together (other opioid, alcohol, Benzodiazepine, Amphetamine or cocaine, Cannabis) and then divided by their size. Then it was rescaled to a percentage scale and those who had a higher than 25% score were categorized as moderate/high and below 25% as low.


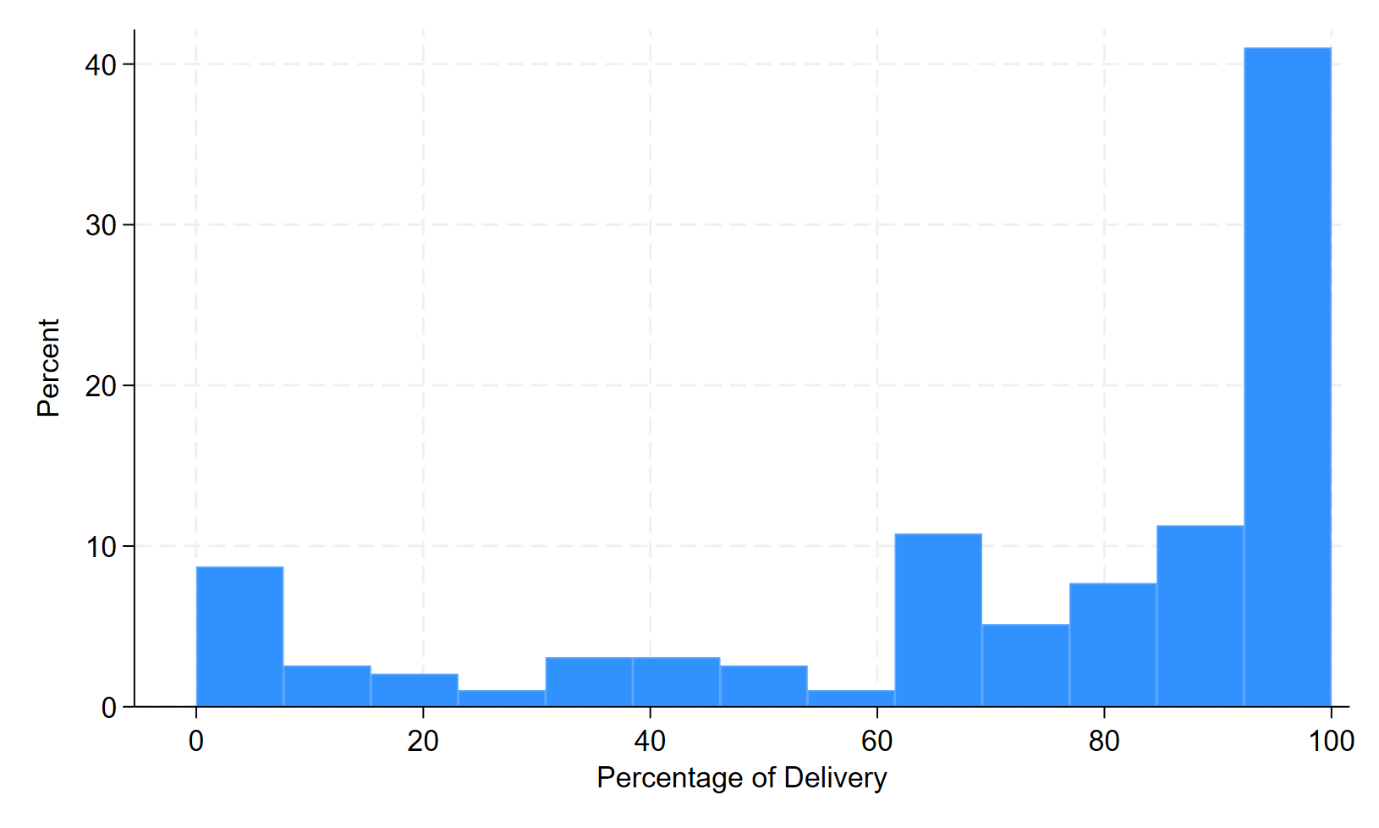


**Figure S1.** Percentage of frequency of delivery of smoothie supplements in OAT clinics for the intervention arm.


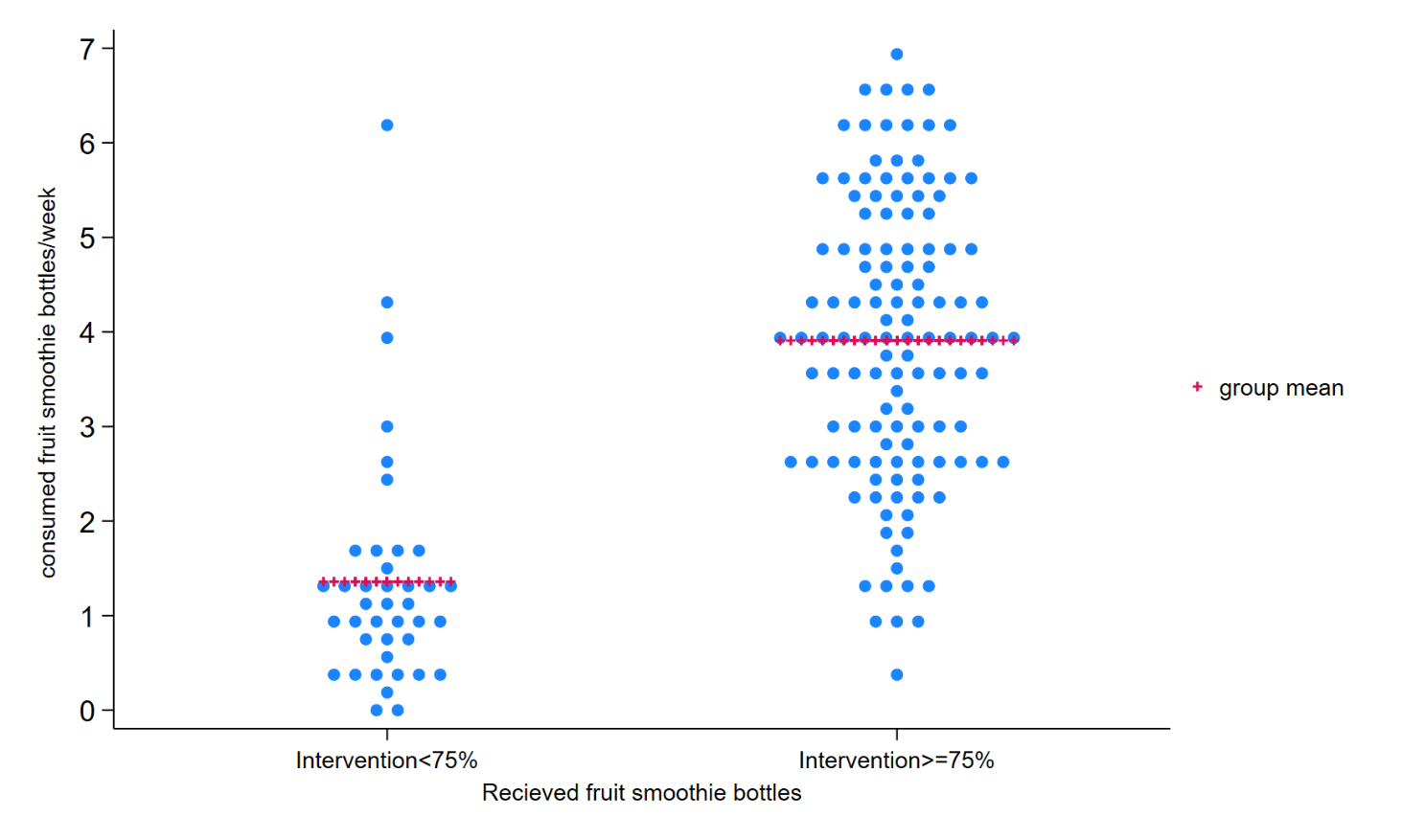


**Figure S2.** Mean number of smoothie bottles consumed per week by the participants in the intervention group in the intervention period.

*Each blue dot represents one participant in the intervention group. The red line represents the mean in each group (Received <75% of the offered smoothies, and >= 75%). The control arm is not presented in this figure, and it is assumed that they have not consumed smoothies.


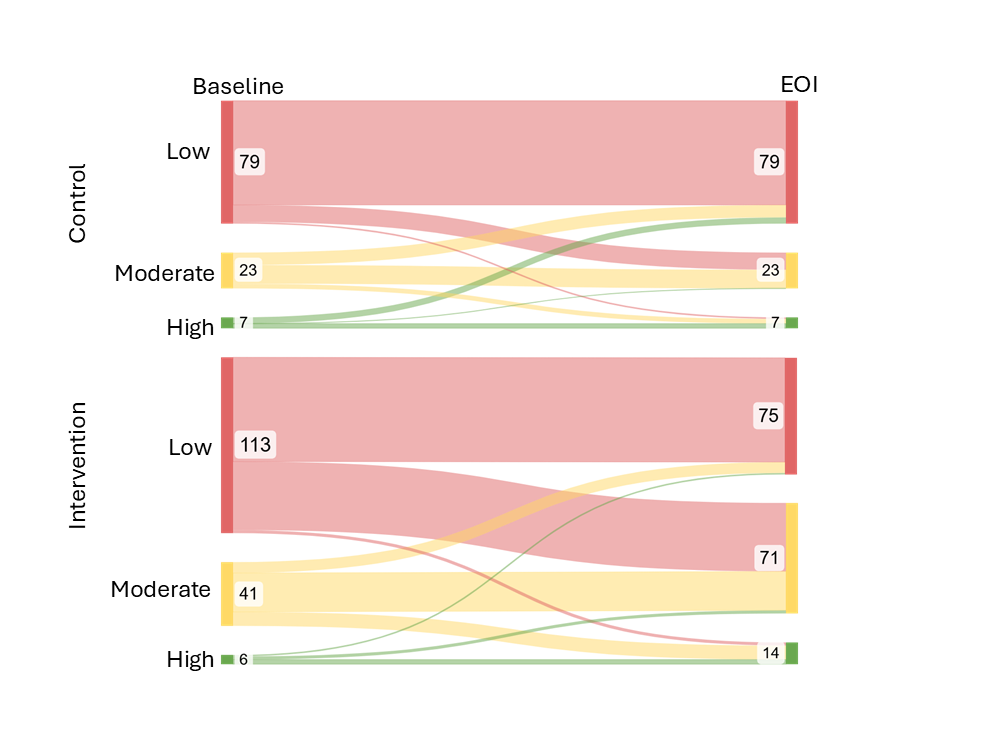


**Figure S3.** Sankey plot presenting the changes in the categories of intake of fruits and vegetables after the intervention. EOI: end of intervention. Participants who had outcome values at the end of the intervention were included in this plot (n=269).

(The Sankey diagram was created using Sankeymatic (http://www.sankeymatic.com/build [May 2025]). )


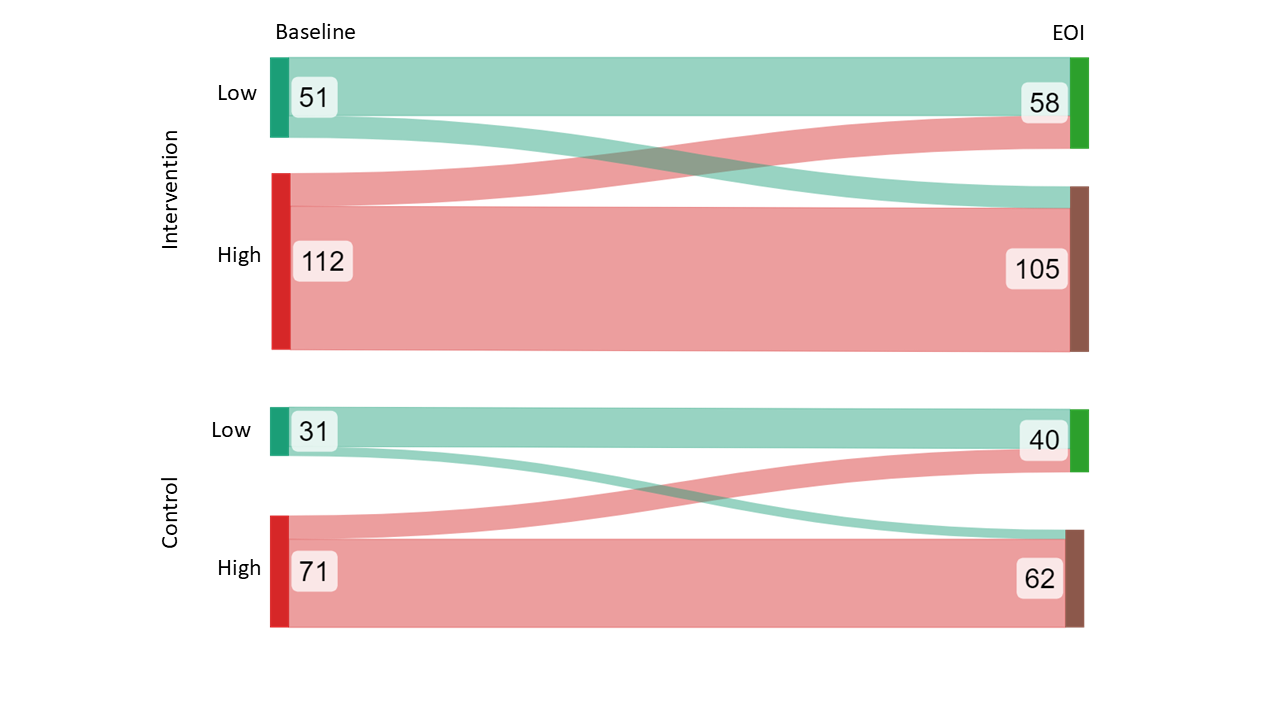


**Figure S4.** Sankey plot presenting the changes in the categories of psychological distress after the intervention. EOI: end of intervention. Participants who had outcome values at the end of the intervention were included in this plot.

(The Sankey diagram was created using Sankeymatic (http://www.sankeymatic.com/build [December 2024]). )


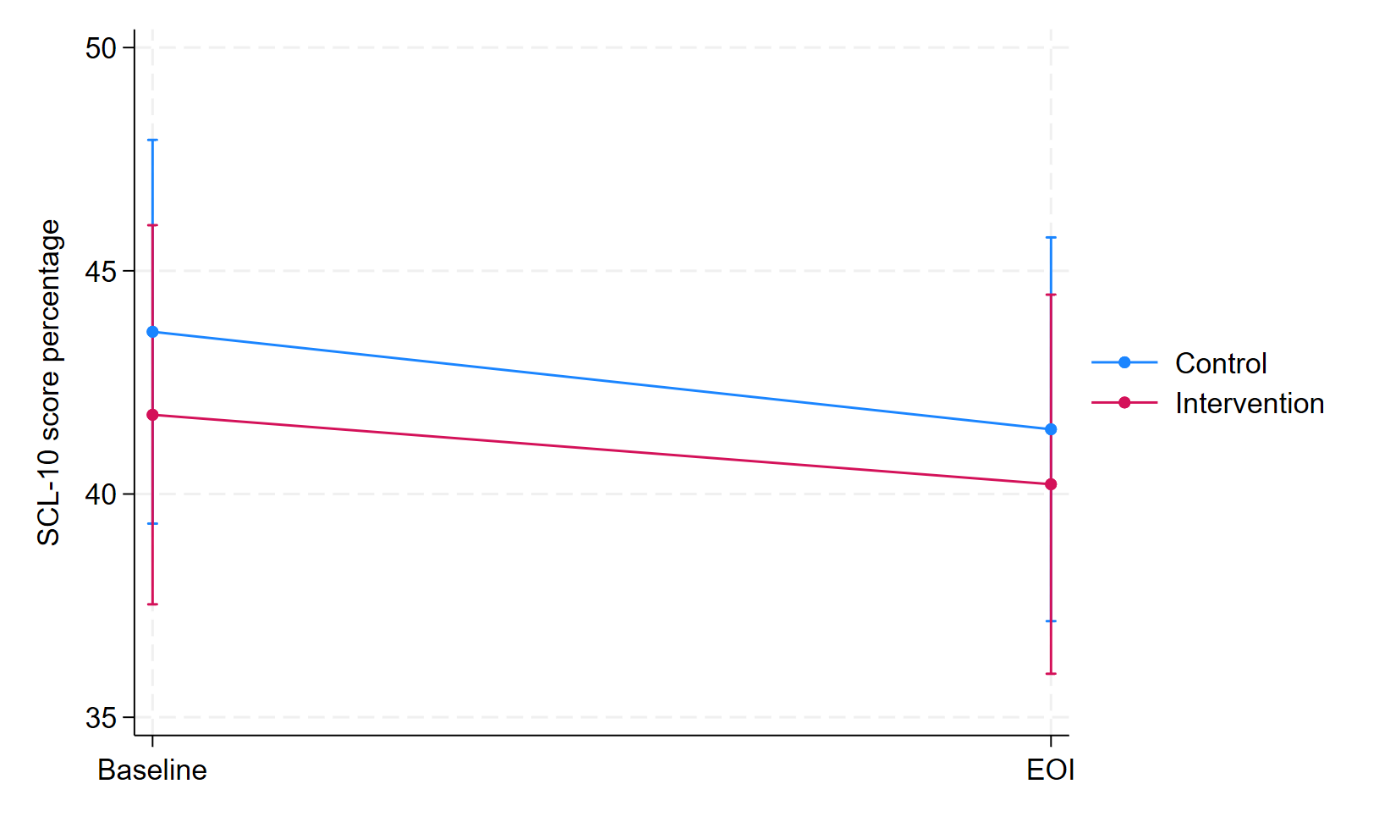


**Figure S5.** Changes in the percentage of SCL-10 after the intervention (Per protocol analysis). EOI: end of intervention


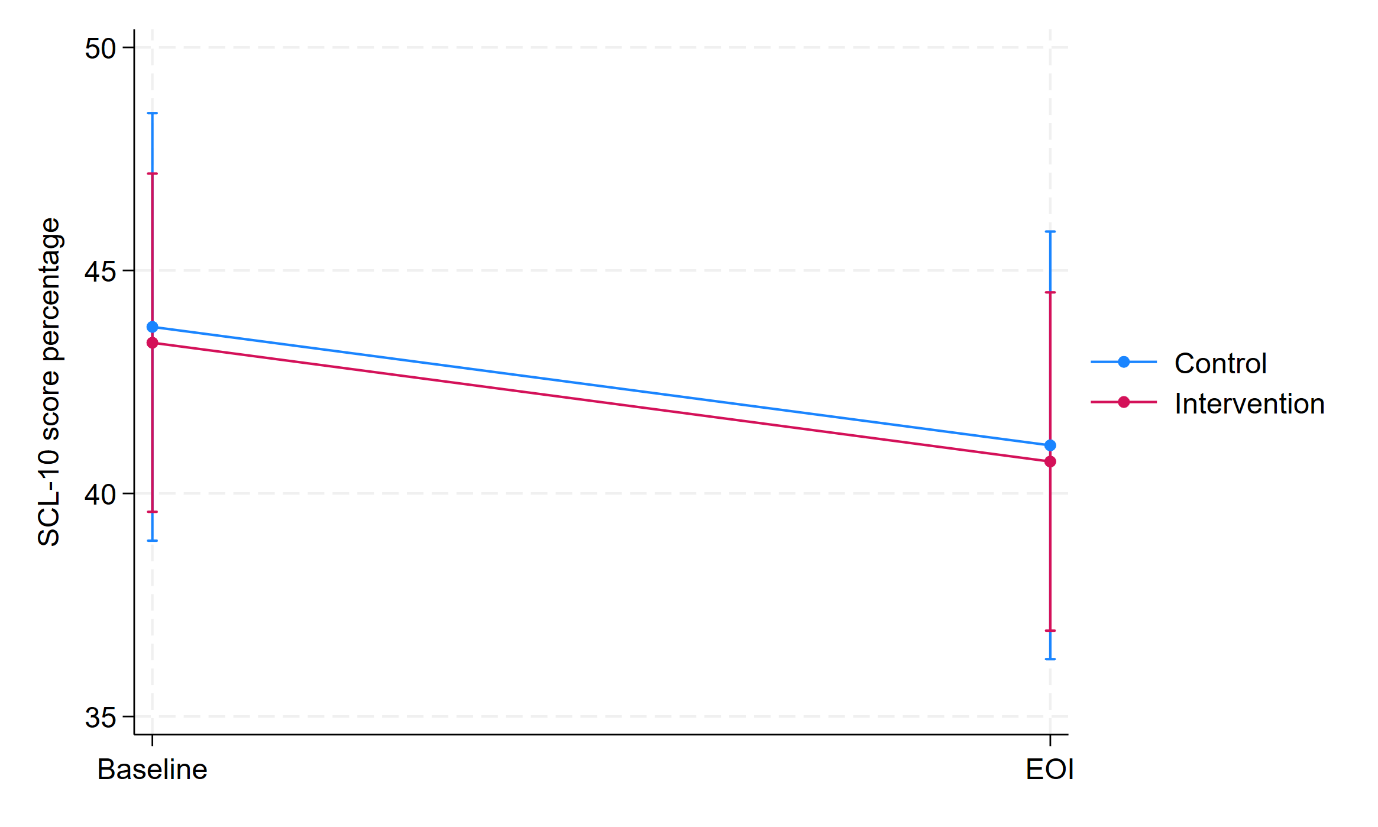


**Figure S6**. Changes in the percentage of SCL-10 after the intervention (Sensitivity analysis of Intention to treat analysis). EOI: end of intervention


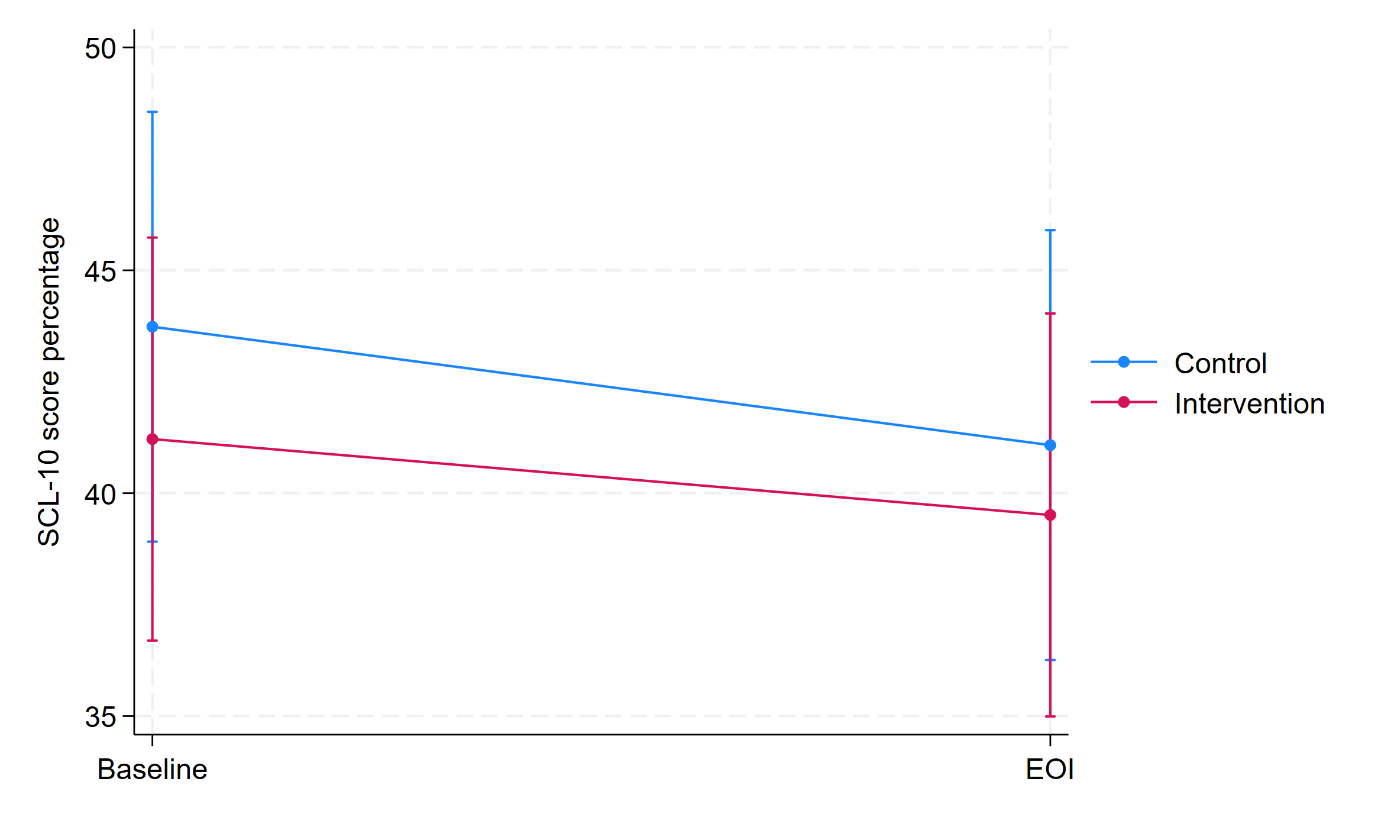


**Figure S7.** Changes in the percentage of SCL-10 after the intervention (Sensitivity analysis of per protocol analysis). EOI: end of intervention


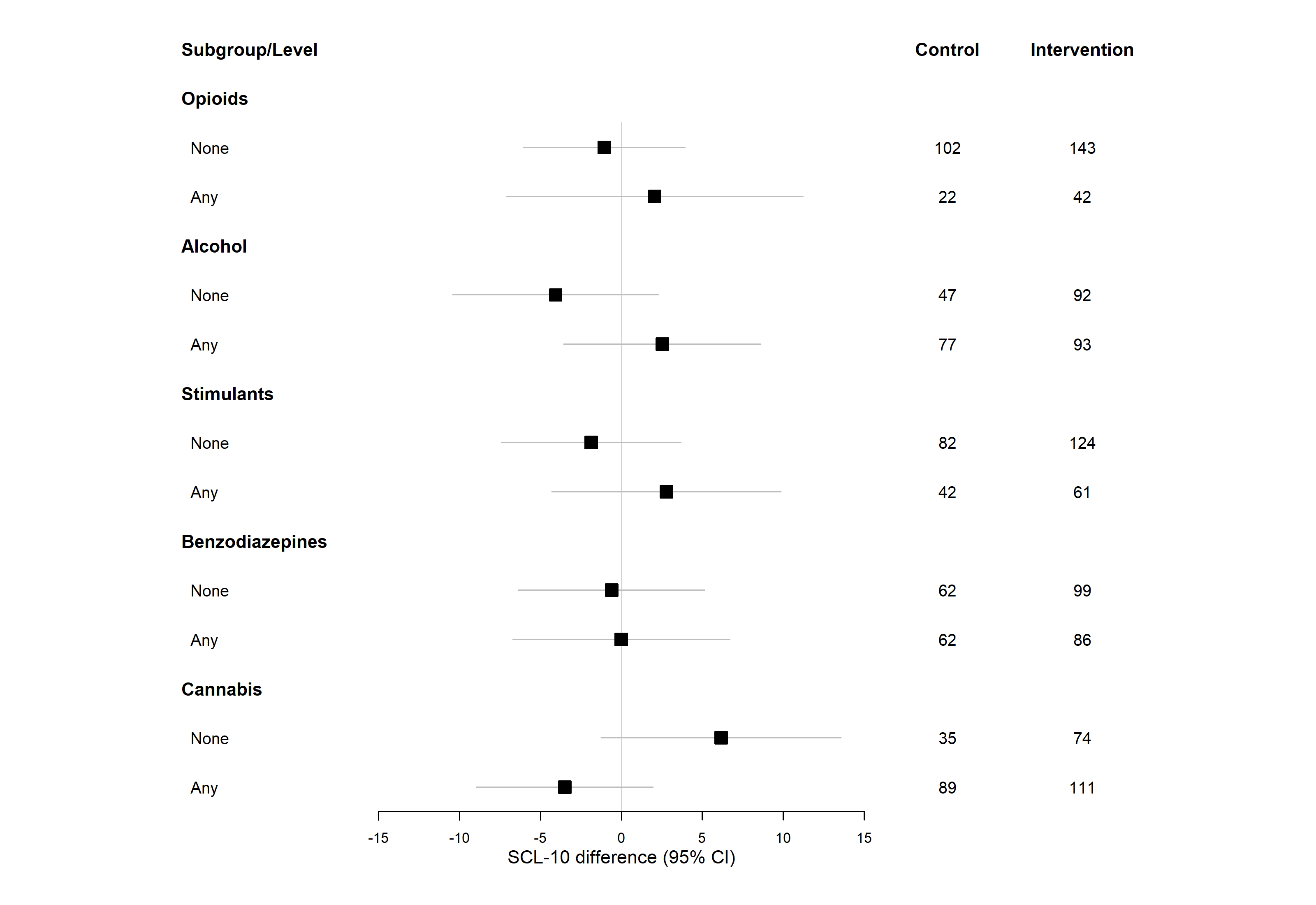


**Figure S8.** Forest plot of subgroup analysis. The difference between arms in changes in the percentage of mean SCL-10, stratified for use of opioids, alcohol, stimulants, benzodiazepines, and cannabis. The changes in SCL-10% were estimated using linear mixed models.


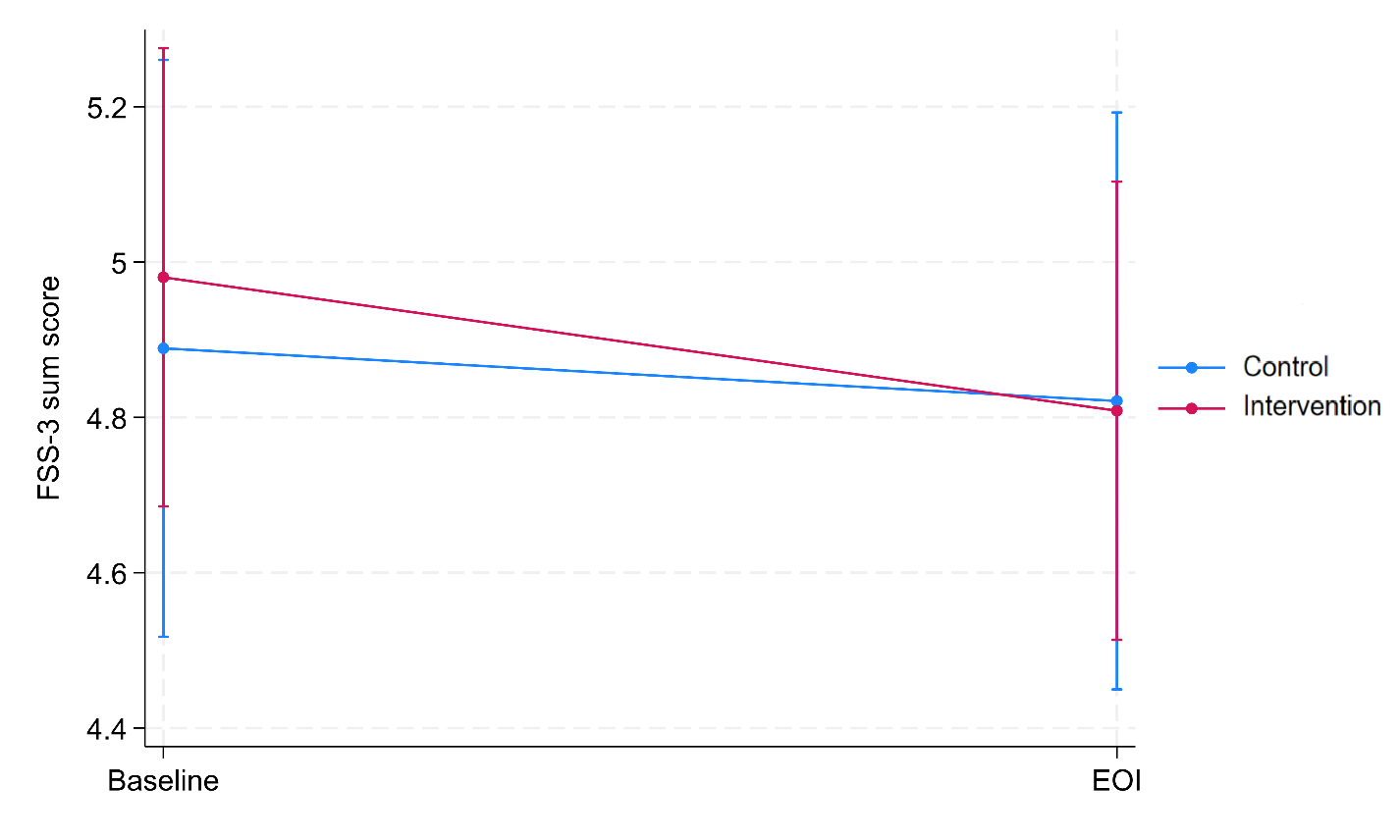


**Figure S9**. Changes in the sum FFS-3 scores from baseline to the end of intervention in intervention and control groups (based on intention-to-treat analysis).


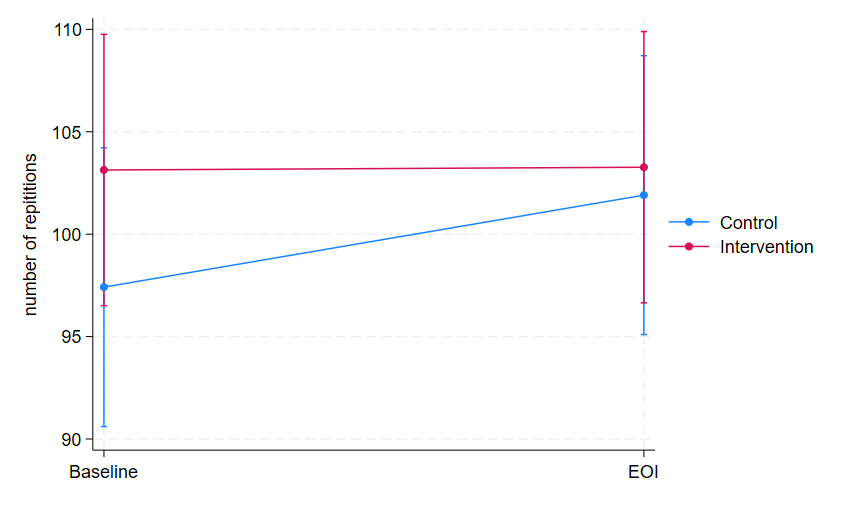


repetitions

**Figure S10**. Changes in the number of repetitions of the 4-minute step test from baseline to the end of intervention in intervention and control groups (based on Intention to treat analysis).


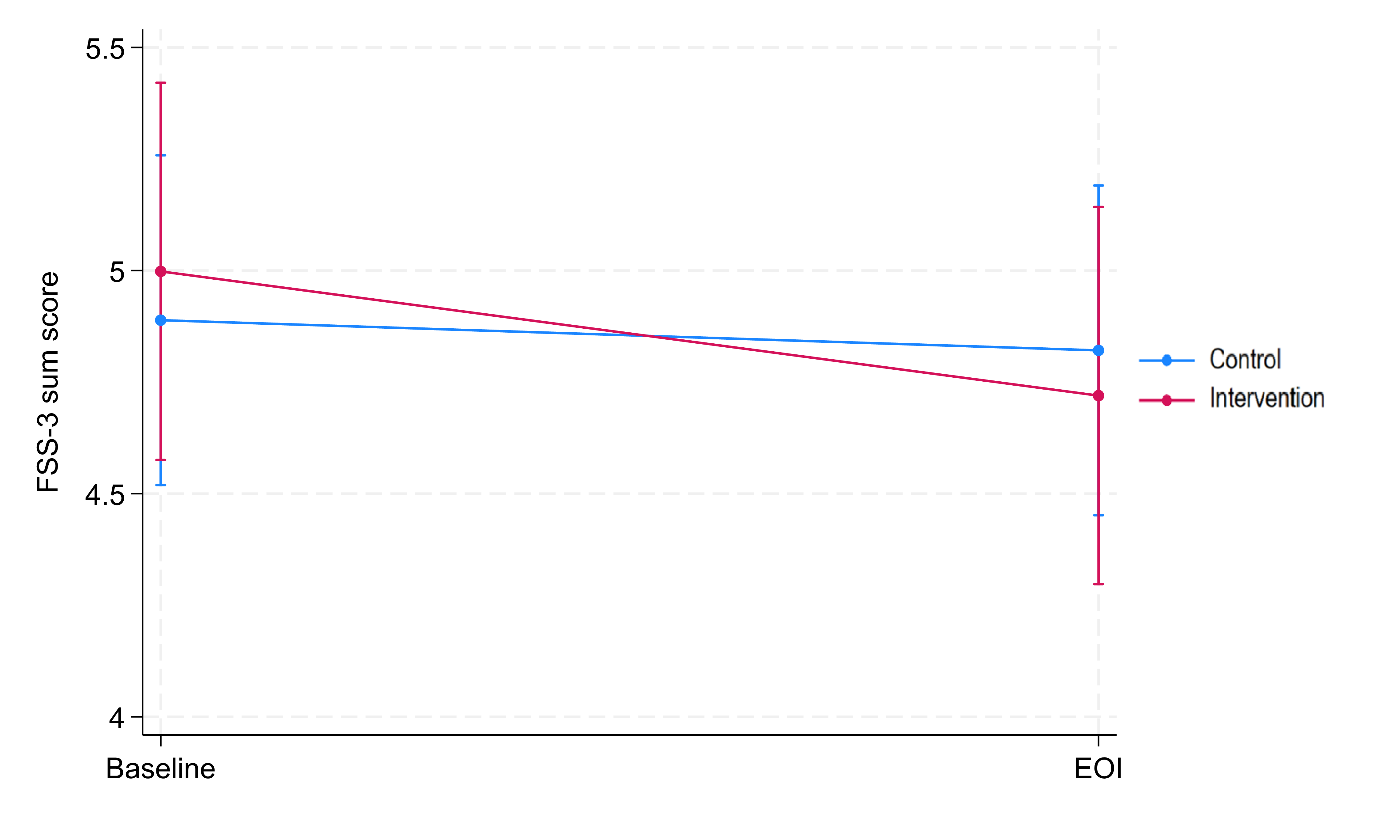


**Figure S11**. Changes in the sum FFS-3 scores from baseline to the end of intervention in intervention and control groups (based on per protocol analysis).


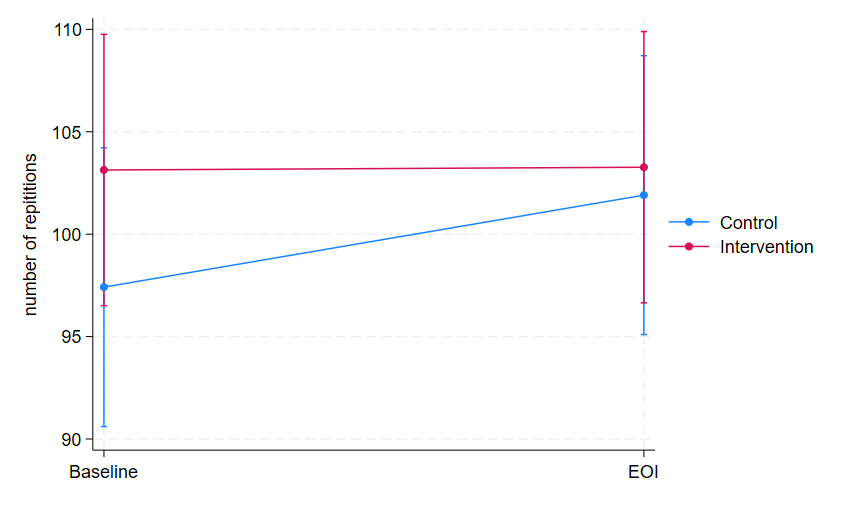


**Figure S12**. Changes in the number of repetitions of the 4-minute step test from baseline to the end of intervention in intervention and control groups (based on Per protocol analysis).

**Table S2:** Baseline values for secondary outcomes

| Variables | Control Baseline  Mean (95 %CI)  or Median (IQR) | Intervention Baseline  Mean (95 %CI)  or Median (IQR) |
| --- | --- | --- |
| Sum FF3-S score (ITT) | 4.9 (4.5; 5.3) | 5.0 (4.7; 5.3) |
| Sum FF3-S score (PP) | 4.9 (4.5; 5.3) | 5.0 (4.6; 5.4) |
| 4m step-test repetitions (ITT) | 97.4 (90.9; 103.9) | 102.6 (97.3; 108.0) |
| 4m step-test repetitions (PP) | 97.4 (90.6; 104.2) | 103.1 (96.5; 109.8) |
| Folate (ITT) | 11 (15) | 12 (14) |
| Folate (PP) | 11 (15) | 13 (15) |
| α-carotene (ITT) | 12 (12) | 9.3 (9.8) |
| α-carotene (PP) | 12 (12) | 9.2 (10) |
| β-carotene (ITT) | 60 (92) | 45 (52) |
| β-carotene (PP) | 60 (92) | 62 (49) |
| Lutein (ITT) | 28 (22) | 23 (22) |
| Lutein (PP) | 28 (22) | 24 (21) |
| Zeaxanthin (ITT) | 8.2 (5.4) | 5.5 (5.9) |
| Zeaxanthin (PP) | 8.2 (5.4) | 5.2 (4.6) |
| β -cryptoxanthin (ITT) | 15 (20) | 14 (15) |
| β -cryptoxanthin (PP) | 15 (20) | 14 (15) |
| Lycopene (ITT) | 85 (74) | 81 (103) |
| Lycopene (PP) | 85 (74) | 80 (83) |
| Total carotenoids (ITT) | 204 (209) | 223 (205) |
| Total carotenoids (PP) | 204 (209) | 233 (184) |

*Levels of biomarkers of intake of fruits and vegetables (nmol/L). Intervention group: ITT: n= 49, PP: n=36; Control group: n=27
